# Vitamin D deficiency in relation with the systemic and central inflammation during multiple sclerosis

**DOI:** 10.5937/jomb0-37676

**Published:** 2023-08-25

**Authors:** Sawsan Feki, Manel Naifar, Mariem Dammak, Sabrina Majdoub, Salma Sakka, Ali Yesmine Ben, Hend Hachicha, Chokri Mhiri, Fatma Ayadi, Hatem Masmoudi

**Affiliations:** 1 University of Sfax, Habib Bourguiba Hospital, Immunology Laboratory, Sfax, Tunisia; 2 University of Sfax, Habib Bourguiba Hospital, Biochemistry Laboratory, Sfax, Tunisia; 3 University of Sfax, Habib Bourguiba Hospital, Neurology Department, Sfax, Tunisia

**Keywords:** multiple sclerosis, vitamin D, inflammatory cytokines, CSF oligoclonal bands, multipla skleroza, vitamin D, inflamatorni citokini, CSF oligoklonske trake

## Abstract

**Background:**

During the last decade, vitamin D (VitD) has become a topic of interest in immune regulation, especially in multiple sclerosis (MS) disease. Amongst the wide range of effects reported for this vitamin on the immune system, a regulatory role on cytokines production has been described. Our aim is to analyze the status of VitD and its correlation with the circulating inflammation and the intrathecal humoral response during MS.

**Methods:**

We analyzed samples of 318 individuals: 108 MS patients and 210 controls. Determination of 25-(OH) VitD3 level in serum was made using electrochemiluminescence method. Circulating inflammatory cytokines (IL-6, IL-8, IL-10, TNF-a, IL12p70 and IL-1b) were investigated using Cytometer Bead Array Technology. The central humoral response was characterized using CSF isofocusing test and IgG Index calculation.

**Results:**

As expected, mean value of VitD was significantly lower in MS group (26 nmol/L) than in control group (34.75 nmol/L) (p=0.002), with a severe deficiency in 67% of MS patients. Mean value of VitD was significantly lower in MS female patients. Regarding cytokines, mean value of TNFa was significantly higher in MS patients with oligoclonal bands of IgG in the CSF. IL6 was positively correlated with IgG level in serum of MS patients.

**Conclusions:**

Our results support the association of VitD deficiency with MS, especially in female patients of our region. However, the vitamin level seems to not correlate with inflammatory cytokines nor with disability. Interestingly, TNFa and IL6 levels were correlated with the intrathecal synthesis of IgG and the circulating IgG level, respectively.

## Abstract (sr)

**Uvod:**

Tokom poslednje decenije, vitamin D (VitD) je postao tema od interesa za regulaciju imuniteta, posebno kod bolesti multiple skleroze (MS). Među širokim spektrom efekata prijavljenih za ovaj vitamin na imuni sistem, opisana je regulatorna uloga u proizvodnji citokina. Naš cilj je bio da analiziramo status VitD i njegovu korelaciju sa cirkulišućom inflamacijom i intratekalnim humoralnim odgovorom tokom MS.

**Metode:**

Analizirali smo uzorke od 318 osoba: 108 pacijenata sa MS i 210 kontrola. Određivanje nivoa 25-(OH) VitD3 u serumu je izvršeno metodom elektrohemiluminiscencije. Cirkulišući inflamatorni citokini (IL-6, IL-8, IL-10, TNF-a, IL12p70 i IL-1b) su ispitivani korišćenjem Citometer Bead Array Technology. Centralni humoralni odgovor je okarakterisan korišćenjem testa izofokusiranja CSF i izračunavanja IgG indeksa.

**Rezultati:**

Kao što se i očekivalo, srednja vrednost VitD je bila značajno niža u grupi sa MS (26 nmol/L) nego u kontrolnoj grupi (34,75 nmol/L) (p=0,002), sa teškim nedostatkom kod 67% pacijenata sa MS. Srednja vrednost VitD bila je značajno niža kod pacijenata sa MS. Što se tiče citokina, srednja vrednost TNFa je bila značajno viša kod pacijenata sa MS sa oligoklonalnim trakama IgG u CSF. IL6 je bio u pozitivnoj korelaciji sa nivoom IgG u serumu pacijenata sa MS.

**Zaključak:**

Naši rezultati podržavaju povezanost deficita VitD sa MS, posebno kod pacijenata našeg regiona. Međutim, čini se da nivo vitamina nije u korelaciji sa inflamatornim citokinima niti sa invaliditetom. Zanimljivo je da su nivoi TNFa i IL6 bili u korelaciji sa intratekalnom sintezom IgG i nivoom IgG u cirkulaciji, respektivno.

## Introduction

Vitamin D (VitD), a fat vitamin obtained from the diet (plant and animal origin) but mostly from skin synthesis after sun (UV) exposure, has become a topic of interest in immune regulation, especially in multiple sclerosis (MS) disease. Many reported findings emphasize the suggested pathogenic role of VitD deficiency in MS. Observational studies concluded a reduced circulating VitD level as a risk factor for developing the disease, and recent experimental studies suggested an effect of this vitamin in neuroprotection and myelin repair [1] [2] [3] [4] [5] [6] [7]. Vitamin D3 (cholecalciferol) is quantitatively the main form and the most bioactive molecule. The molecule is transported to the liver, to be hydroxylated to 25 hydroxyvitamin D3 (25 (OH) D3) which serum level reflects the VitD status. In the kidney, another hydroxylation in a second position leads to 1,25 (OH)2 D, which is the most bioactive metabolite. CYP27B1 (25(OH)D3-1α-hydroxylase), the enzyme responsible for the synthesis of this active metabolite, is expressed in immune cells [4] [5] [6]. Among the wide range of VitD effects on the immune system, it has been reported that there is a regulatory role for this vitamin on cytokine production [3] [6] [8] [9]. During MS, an inflammatory demyelinating disease of the central nervous system (CNS), inflammatory cytokines are essential for the regulation of leucocyte trafficking across the blood-brain barrier and for the evolution of MS lesions. This leukocyte infiltration of the CNS causes inflammation, demyelination, and subsequent characteristic disorders (sclerotic plaques) [10] [11]. In addition, it has been proven that active MS lesions involve cellular effectors (T-cell and macro phage infiltration) and immune mediators (chemo kines, cytokines, and adhesion molecules) [12]. Profiling cytokines in MS may therefore improve knowledge about immune pathways involved in the development of the CNS lesions, and in monitoring the disease course and therapy responses. It may also help to identify new cytokines-related therapies [11] [12] [13].

In our study, we aim to evaluate VitD status in Tunisian MS patients in comparison with healthy controls and to find a correlation of the level of this vitamin with the peripheral (profile of 6 inflammatory cytokines) and central inflammation (intrathecal humoral immune response reported as the hallmark of the disease) during MS conditions.

## Materials and methods

### Study population

In this study, we analyzed serum samples (peripheral blood) of 318 individuals: 108 South Tunisian patients with definite primo-diagnosis of MS according to McDonald criteria (2010) and 210 healthy controls, matched in geographic origin, sex, and age to MS patients.

In our routine practice, samples (couples of cerebrospinal fluid (CSF) and serum) of patients with suspicion of inflammatory disorder of the SNC are addressed from the Neurology Department to our Laboratory for biological investigation [14]: CSF isofocusing (to detect oligoclonal bands (OCB) of Immunoglobulin G (IgG) in the CSF) and determination of total IgG and albumin levels in sera and CSF for the calculation of *Tibbling et Link* IgG Index [15]. These 2 routine tests are used in such context, to detect an intrathecal (within the CNS) synthesis of IgG (revealed as positive OCB in CSF and/or IgG index >0.7), which is considered the hallmark of MS disease.

Demographic, clinical, radiological, and therapeutic features of patients are obtained from medical records.

In this serological study, we included a well-defined MS group from our database, which were newly diagnosed at the time of sampling (Mc Donald Criteria 2010). Patients were also chosen according to the period of sampling (spring-summer of each year). A supplementation on VitD and/or a corticosteroid or an immunosuppressive treatment during the month before the sampling were considered exclusion criteria. MS Patients with additional autoimmune or inflammatory diseases were also excluded.

This MS cohort (n=108) was composed of 77 women and 31 men, with a mean age of 36 years old (19–59 years). It comprises 87 patients with Relapsing-remitting MS (RR-MS) and 21 with progressive form (no other inflammatory, autoimmune or infectious diseases). The clinical evaluation of disability was made using the Expanded Disability Status Scale (EDSS) at the time of collecting samples. According to CSF Isofocusing results, 89 MS patients were OCB positive and 19 were OCB negative. All patients benefited from a longitudinal follow-up for at least 2 years. The clinical and biological features of our MS cohort are summarized in Table 1.

**Table 1:** Clinical, radiological and biological features of enrolled patients and control groups *MRI: Magnetic resonance imaging; **EDSS: Expanded Disability Status Scale, ***CSF: cerebrospinal fluid; ****OCB: Oligoclonal bands

	MS group	Control group
Size	n=108	N=210
Age (years)<br>[Mean (range)]	36 (20–59)	35 (20–56)
Gender	77 F / 31 M	145 F/65 M
Diagnosis	MS	Healthy<br>individuals
Clinical form (at sampling)<br>Relapsing-remitting<br>Progressive	87<br>21	
Optical Neuritis	45	
MRI*<br>Cerebral Abnormalities<br>Myelitis	108<br>68	
EDSS** Mean [Mean<br>(SD; range)]<br>(at sampling)	2.8 (1.86; 0–7)	
IgG-serum level [Mean<br>(range)] (g/L)	11.3 (5–15.8)	
Total IgG Index [Mean(range)]	1.1 (0.41–3.27)	
CSF*** Electro-isofocusing<br>OCB**** (+)<br>OCB (-)	89<br>19	
Treatment<br>Interféron β-1b<br>Need for Natalizumab	91<br>17	

Regarding the control group, it comprises 145 women and 65 men, with a mean age of 35 years old (20–56 years). The study was approved by the Local Ethics Committee.

### Determination of vitamin D level in serum of MS patients and healthy controls

In our study, the serum of the MS group (n=108) and healthy controls (n=210) was analyzed to determine levels of VitD (25-hydroxyvitamin D3) using the electrochemiluminescence method (Cobas 6000, Roche® ; Switzerland; Normal value ≥ 75 nmol/L). Individuals with abnormal levels were classified as insufficient (vitamin D level <75 nmol/L) or deficient (vitamin D level <50 nmol/L) in VitD. A level < 25 nmol/L was considered a severe deficiency (According to the classification proposed by Holick MF [16]).

### Determination of inflammatory cytokines levels in serum of MS patients

Among our cohort of MS patients (n=108), 55 patients were investigated for the determination of 6 inflammatory cytokines levels in their serum. This subgroup comprises 41 oligoclonal bands (OCB) positive and 14 OCB negative MS patients (45 relapsing-remitting and 10 progressive MS) (Table 2).

**Table 2:** Characteristics of MS subgroup enrolled in the determination of levels of inflammatory cytokines in serum ^*^MRI: Magnetic resonance imaging; ^**^EDSS: Expanded Disability Status Scale, ^***^CSF: cerebrospinal fluid; ^****^OCB: Oligoclonal bands

MS subgroup	
Size	n=55
Age (years) [Mean (range)]	36 (20–59)
Gender	42 F / 13 M
Diagnosis	MS
Clinical form<br>Relapsing-remitting<br>Progressive	45<br>10
Optical Neuritis	25
MRI^*^<br>Cerebral Abnormalities<br>Myelitis	55<br>29
EDSS^**^ Mean [Mean (SD; range)]	2.6 (1.7; 0–6.5)
Total IgG Index [Mean (range)]	1.1 (0.4–2.8)
IgG-serum level [Mean (range)] (g/L)	11.2 (7.2–15.8)
CSF^***^ Electro-isofocusing<br>OCB^****^ (+)<br>OCB (-)	41<br>14
Treatment<br>Interféron β-1b<br>Need for Natalizumab	50<br>5

A commercial kit (Human Inflammatory Cytokines CBA Kit, BD Biosciences®, USA) was used to measure levels of 6 cytokines in serum: IL-6, IL-8, IL-10, TNF-α, IL12p70, IL-1β by Cytometer Bead Array (CBA) Technology. The kit contains six bead populations with distinct fluorescence intensities coated with capture antibodies specific for the 6 cytokine proteins. Each capture bead in the array has unique fluorescence intensity and is coated with a capture antibody specific for a single analyte. A combination of different beads is mixed with a sample or standard and a mixture of detection antibodies that are conjugated to a reporter molecule (Phycoerythrin PE). Following incubation and subsequent washing, data were acquired on a BD FACS Canto™ II flow cytometer(BD Biosciences®, USA). All standards and samples were measured in duplicate. FCAP Array™ software was used to generate results in graphical and tabular format.

### Statistical analysis

Statistical analysis was performed using SPSS 20.0 software. Normality of distribution was checked using Kolmogorov–Smirnov test (K–S test). Quantitative variables were expressed with mean and range values, the statistical significance of differences between groups of patients was assessed by the Mann-Whitney U test. A Levene's Test was run to check the equality of variances. Qualitative variables were tested using Fisher's exact test.

Correlations were studied using the Spearman test. A *p*-value of < 0.05 was defined as statistically significant. Correlation studies were made using R Software.

## Results

In our study, we investigated the serum levels of 25 (OH) VitD in a Tunisian MS cohort (n=108) in comparison to a healthy control group (n=210), matched in geographic, sex, and age to the patients. In a second step, we evaluated 6 circulating inflammatory cytokines (IL-6, IL-8, IL-10, TNF-α, IL12p70, IL-1β) levels in the serum of a subgroup of MS patients (n=55) to study their correlation with the corresponding VitD level. Then, we performed a correlation study of the analyzed blood markers with the demographic, clinical, radiological, and biological features of MS patients.

### Association of 25 (OH) vitamin D deficiency with MS and correlation with patients' characteristics

**Figure 1:**
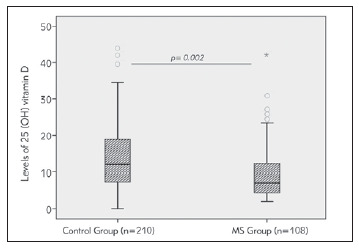
Box plot showing the comparative distribution of circulating 25(OH) vitamin D levels in patients with multiple sclerosis (MS group; n=108) and healthy controls (Control group; n=210). The median value of vitamin D is significantly lower in the MS group in comparison with the control group (p=0.002)

The distribution of circulating 25(OH) VitD levels in MS patients and healthy controls are shown in Figure 1.

**Figure 2:**
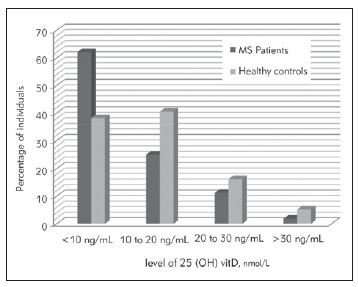
Bar graph illustrating the qualitative distribution of 25 (OH) Vitamin D status in the MS group (n=108) and controls (n=210). Normal value (> 75 nmol/L);<br>Insufficiency (vitamin D level < 75 nmol/L); Deficiency (vitamin D level < 50 nmol/L) or Severe deficiency (<25 nmol/L)

The quantitative analysis, shown in Table 3, revealed that the mean value of this vitamin was significantly lower in the MS group (26 nmol/L) in comparison with the control group (34.75 nmol/L) (p=0.002). This significant difference was preserved between the MS group and controls upon gender clustering of the 2 groups (Table 3). Regression analysis demonstrated that the association of low levels of VitD with MS disease was independent of age and gender. Figure 2

**Table 3:** Quantitative analysis of 25 (OH) vitD levels in MS patients and controls

	25 (OH) Vitamin D levels [Mean (SD)]			p-value
MS patients versus				
Disease	nmol/L	MS group<br>(n=108)<br>26 (24)	Healthy Controls<br>(n=210)<br>34.75 (21.1)	0.002
Gender	nmol/L	Female patients<br>(n=77)<br>18.75 (13.75)	Female controls<br>(n=145)<br>25.05 (15.25)	0.003
	nmol/L	Male patients<br>(n=31)<br>44.5 (33.25)	Male Controls<br>(n=65)<br>56.15 (15.75)	0.023
MS subgroups				
Gender	nmol/L	Female patients<br>(n=77)<br>18.75 (13.75)	Male patients<br>(n=31)<br>44.5 (33.25)	<0.001
MS clinical form	nmol/L	RR-MS<br>24.02 (20)	progressive MS<br>25.65 (16.75)	0.76
IgG Index	nmol/L	Low (<0.7)<br>29 (23.25)	High (>0.7)<br>22.8 (16.75)	0.26
OCB profile in CSF	nmol/L	OCB Positive MS<br>24.65 (20.25)	OCB Negative MS<br>23 (15.25)	0.71
Intrathecal Synthesis<br>of IgG	nmol/L	Positive<br>22.5 (16.75)	Negative<br>29.9 (23.5)	0.19
Needed Treatment	nmol/L	Interféron β-1b26.9 (25.5)	Natalizumab<br>22.75 (16.5)	0.45

Among MS patients, the mean value of VitD was significantly lower in the female subgroup (18.75nmol/L) in comparison with males (44.5 nmol/L). There was no other significant difference in VitDlevels between MS subgroups regarding the clinical form of the disease (RR/ progressive), the biological parameters (IgG Index high/low, OCB positive/negative in CSF, Intrathecal Synthesis of IgG positive/negative) and the treatment administrated (Interferon β-1b/ Natalizumab). The quantitative analysis of levels of VitD and other parameters (IgG Index, serum IgG levels, Expanded Disability Status Scale (EDSS)) did not reveal any correlation.

We performed a qualitative analysis of VitD status in patients and controls enrolled in this study. Individuals with abnormal levels were classified as insufficient (level < 75 nmol/L) or deficient (50 nmol/L) in VitD. A level below 25 nmol/L was considered a severe deficiency. Regarding the MS group, the majority (87%) of patients had VitD deficiency (VitD level < 50 nmol/L). About two-thirds of the group (64.8%) had a severe deficiency in this vitamin (level < 25 nmol/L). This percentage was significantly higher than in controls (34.5%) (p<0.001). The association of 25 OH VitD severe deficiency to MS disease did not disappear upon gender clustering of patients and control groups.

### Study of the circulating inflammatory cytokines levels and their correlation with Vitamin D levels and patients’ characteristics

### Distribution of the circulating inflammatory cytokines levels in the study MS population

In the second step of our study, we determined the levels of 6 circulating inflammatory cytokines (IL-6, IL-8, IL-10, TNF-α, IL12p70, IL-1β) levels in the serum of a subgroup of MS patients (n=55) by a multiplex cytometric technique. Values (mean, standard deviation, minimum and maximum) relative to each cytokine measured in MS patients are summarized in Table 4.

**Table 4:** Results of inflammatory cytokines measurements in serum of patients with multiple sclerosis disease

Cytokine	Mean +/- SD<br>(pg/mL)	Min-Max<br>(pg/mL)
IL6	4.1+/-2.1	2.5–11.7
TNF	13.8+/-11.8	4.5–65.2
IL10	4.1+/-5	3.3–14.8
IL8	9.6+/-9.8	3.7–48
IL1b	8.6+/-3.3	7.3–20.4
IL12p70	2.6+/-1.2	1.9–5.4

### Circulating inflammatory cytokines levels and OCB detection in MS

**Figure 3:**
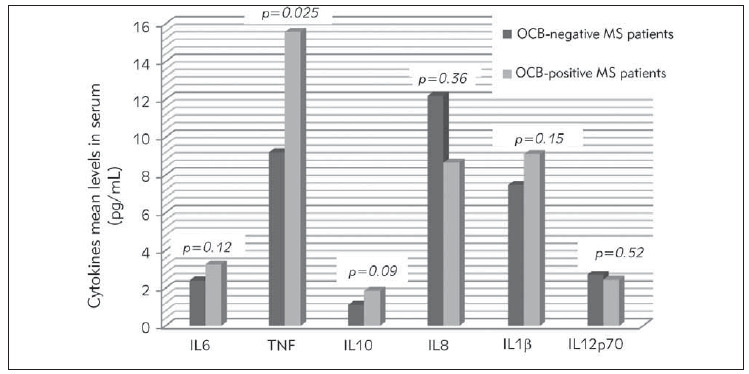
Bar graph showing comparative mean values of circulating inflammatory cytokines in oligoclonal bands (OCB)-positive (n=41) Versus OCB negative MS patients (n=14). Mean values of IL6, TNF, IL10, and IL1b were higher in OCB positive than in OCB negative patients, in contrary to IL8 and IL12p70 which means were lower in the case of OCB positivity. The difference reached a statistical significance only for TNF (OCB positive (15.57 pg/mL) Vs OCB negative patients (9.2 pg/mL); p=0.025)

The MS subgroup tested for cytokines comprises 14 OCB negative and 41 OCB positive patients. Mean values of IL6, TNF, IL10, and IL1β were higher in OCB positive than in OCB negative patients, in contrary to IL8 and IL12p70 which means were lower in the case of OCB positivity (Figure 3). The difference reached a statistical significance only for TNF. The mean value of this cytokine was significantly higher in OCB positive (15.57 pg/mL) than in OCB negative patients (9.2 pg/mL) (Figure 3).

There was no other significant difference in mean values of cytokines between subgroups of patients regarding the other qualitative parameters (Age, gender, clinical form, MRI features, treatment).

### Correlation study between the circulating inflammatory cytokines and quantitative parameters measured in serum of MS patients

Results of the correlation study between the levels of the 6 studied inflammatory cytokines and the other quantitative parameters (25 (OH) VitD level, IgG Index, IgG level) in serum of MS patients is shown in Table 5.

**Table 5:** Correlation study between levels of inflammatory cytokines, 25 (OH) vitD3, and other quantitative parameters in the MS group

Correlation		25 OH VitD	IgG Index	IgG level<br>in serum	IL6	TNF	IL10	IL8	IL1 β
25 OH VitD	R								
	*P*								
IgG Index	R	-0.075							
	P	0.555							
IgG level<br>in serum	R	-0.068	0.115						
	P	0.505	0.338						
IL6	R	-0.051	0.157	0.304					
	P	0.746	0.285	0.035^*^					
TNF	R	-0.099	0.060	0.270	0.402*				
	P	0.535	0.684	0.060	0.005				
IL10	R	0.036	0.220	0.111	0.630^*^	0.394^*^			
	P	0.824	0.134	0.452	<0.001	0.006			
IL8	R	0.107	0.133	0.089	0.646*	0.130	0.534^*^		
	P	0.501	0.368	0.549	<0.001	0.377	<0.001		
IL1 β	R	-0.212	0.172	0.145	0.615^*^	0.496^*^	0.585^*^	0.239	
	P	0.177	0.238	0.321	<0.001	<0.001	<0.001	0.102	
IL12p70	R	-0.134	-0.050	0.152	0.510^*^	0.451^*^	0.495^*^	0.257	0.658^*^
	P	0.397	0.732	0.298	<0.001	0.001	<0.001	0.078	<0.001

The statistical analysis did not reveal any correlation between the studied cytokines and the 25 (OH) VitD level in the serum of MS patients. IL6 was positively correlated with the levels of the 5 other studied inflammatory cytokines (TNF, IL10, IL8, IL12p70, and IL-1β) and with the level of IgG in [the serum of MS patients. Regarding the clinical disability in MS patients, there was no correlation between any of the circulating cytokines with EDSS score in our cohort.

## Discussion

In addition to its well-defined effect on calcium homeo stasis, VitD became a topic of interest in immune regulation, especially in the context of MS [17].

In our study, we analyzed levels of VitD and 6 inflammatory cytokines in the serum of a group of South Tunisian patients with a definite diagnosis of MS and a group of healthy controls. Our main findings support the association of VitD deficiency with MS disease, especially in female patients, and the correlation of circulating levels of TNFα and IL6 cytokines with the intrathecal synthesis of IgG and circulating IgG levels, respectively. However, no correlation was detected between VitD level and the studied cytokines in MS patients.

### Vitamin D status and MS risk and progression

Overall, levels of 25 (OH) VitD in blood were significantly lower in our MS patients than in controls. The majority (87%) of MS patients had VitD deficiency (VitD level <50 nmol/L) and about two-thirds of the group (64.8%) had a severe deficiency in this vitamin (level <25 nmol/L, which was significantly higher than in controls. Despite differences in sampling methodology, several studies performed in different populations replicated the finding that blood levels of VitD are qualitatively and quantitatively lower in MS patients than in healthy controls [4] [18] [19] [20] [21] [22] [23] [24] [25]. However, it has been suggested that this association of VitD status with MS risk may change according to gender and race. In an American prospective case-control study [26], authors examined VitD status in a large cohort of subjects before disease onset. Interestingly, it has been found that VitD deficiency was associated with the development of MS only in whites, but not in blacks and Hispanics. Regarding gender, a Netherlandish study revealed an association of VitD deficiency with MS only in women (but not in men) [27]. In our study, upon gender clustering of the two groups, the quantitative and qualitative association »VitD deficiency-MS disease« did not disappear. However, in the MS group, the mean value of this vitamin was significantly lower in the female subgroup than in the male, which is consistent with the results of another North African study [28]. This finding could be explained by gender-specific differences in VitD metabolism [29] and could also be in relation with religious and cultural factors in our region (gender difference in habits, occupation…), which leads to gender imbalance in sun exposure and therefore, to a different level of skin synthesis of the vitamin. Indeed, sunlight-related VitD synthesis has been suggested to affect MS risk and prevalence. A recent meta-analysis demonstrated that MS is more prevalent in higher than in lower latitudes (differences in sunlight intensity) and hence supported a latitude gradient effect on disease prevalence [30]. Body exposure to sunlight (UV), especially during childhood and adolescence, was reported to be associated with a decreased susceptibility to VitD deficiency and a low MS risk [31] [32] [33] [34] [35] [36]. Furthermore, a relation between the disease risk and the month of birth has been suggested (sunlight exposure during pregnancy) [37]. However, sunlight's effect on MS risk could be related to the immunosuppressive effect of sunlight itself [38]. Therefore, studies evaluating whether VitD deficiency is a risk factor independent of sun exposure are needed. On the other hand, it has been reported that low levels of VitD intake lead to a higher risk of developing MS [39] [40]. It has been particularly demonstrated that VitD supplementation (during 6 months) improves VitD status in correlation with an increase in TGF-β1, which is predictive of protection against MS development [41]. In addition to this potential role in MS risk, it has been reported a possible effect of VitD on the course and clinical severity of the disease. It has been particularly shown that in clinically isolated syndrome patients, a deficient level of this vitamin was a predictor of developing clinically definite MS [42]. Furthermore, during the disease course, higher levels of VitD were associated with a lower MS relapse rate [43] [44] [45] [46]. It has been particularly reported that, during Natalizumab treatment, patients with a deficient level of VitD seem to experience more relapses than patients without deficiency in this vitamin [47]. Regarding disability, many studies argue for an association between a higher level of VitD and less disability evaluated by the EDSS score [46] [21] [44] [45] [48] [49] [50] [51] [52]. However, given that MS patients with severe disability receive less sun exposure, which may result in VitD deficiency, randomized controlled trials are needed to establish the real effects of VitD on MS activity and severity. Indeed, our findings did not reveal any correlation between the level of VitD and the clinical disability evaluated in our patients.

The possible explanation for the described association hypovitaminosis D-MS could either be the immunomodulatory effect of this vitamin on the immune system and/or its special beneficial effects on the nervous system [7]. Indeed, the immune system seems to be an important target of VitD and it has been reported that there is a likely relation between the development and progression of immune disorders especially autoimmune diseases (MS, inflammatory bowel disease, type-1-diabetes), and VitD status and treatment in experimental models, and human. These disorders seem to be very sensitive to molecule availability [7]. The biological basis for this regulation seems to be related to the expression of VitD receptor (VDR) and activating enzyme (cytochrome P450 protein, CYP27B1) in cells that are effectors of immune / inflammatory systems (T and B lymphocytes, macrophages, monocytes, and dendritic cells) [3]. In T cells, VDR expression is upregulated by activation, whereas, in macrophages and dendritic cells, there is a constitutive VDR expression. The active form of VitD could impact its own effects by increasing the expression of VDR and CYP27B1 in some targeted immune cells (macrophages and monocytes). The active form of VitD could therefore play an important role in the regulation of the immune response: inducing monocyte proliferation, expression of IL1 and anti-microbial peptide by macrophage, decrease in dendritic cell maturation and IL12 production, reduction in T cell production of cytokines (IL-2, IL-17, and IFN) and proliferation, promotion of (FOXP3) + regulatory T cells and IL-10-producing T regulatory type 1 (TR1) cells. In addition, 1,25(OH) VitD inhibits the proliferation of B cells and blocks plasma cell differentiation and the production of immunoglobulin [53]. Regarding the nervous system, it has been demonstrated that VDR and 1α hydroxylase are present in the CNS compartment of mammals, including humans [54]. There is accumulating evidence (in vitro, animal, and epidemiological studies) that VitD deficiency may contribute to the risk of neuropsychiatric disorders and neuroin ammatory diseases [6] [55] [56]. In MS disease, it has been suggested that VitD could regulate myelin production by its effect on the oligodendrocyte and different neuronal mechanisms [57]. Regarding MS genetics, the expression of HLA-DRB1*15:01, the major genetic predictor of MS risk, appears to be regulated by VitD. Indeed, VDR-binding elements have been identified in many established MS-associated genes [58].

### Study of circulating inflammatory cytokines in MS patients

In the second step of our study, using a cytometric multiplexing assay, we investigate the circulating levels of 6 cytokines, mostly monokines (IL-6, IL-8, TNF-α, IL-1β) produced by monocytes and macrophages, but also pro-inflammatory lymphocyte T helper (Th)1 (IL12p70) and anti-inflammatory lymphocyte Th2 (IL-10) related cytokines in a subgroup of MS patients (n=55). Overall, our findings showed that mean levels of the studied cytokines in MS were comparative with the levels reported in other MS studies, where authors were interested in profiling cytokines in serum (and/ or CSF) [13], excepted the anti-inflammatory cytokine IL10 which was slightly lower in our study. This result could be partly explained by the reported differences in performance of techniques used in each study (ELISA, electrochemiluminescence assay, or multiplexed immunoassay such as CBA, multiplexed fluorescent bead-based immunoassay) [59], and by the heterogeneity in patient's characteristics (size, stage of the disease, ethnicity). In general, during MS, most of the circulating levels of inflammatory and anti-inflammatory cytokines in serum are reported to be significantly higher than in healthy controls [15] [11] [60], but these levels could be lower than during other inflammatory conditions of the CNS, such as Neuromyelitis Optica [61]. The increase in pro-inflammatory and down-regulatory cytokines in MS could be explained by the pathologic features of the disease: In fact, during the progression of the disease, inflammatory and restorative processes may occur simultaneously in the CNS [13].

In this study, we also aimed to analyze the quantitative correlation of the inflammatory cytokines with the circulating 25 OH VitD and with other biological features of the MS subgroup (Ig Index, IgG level in serum, OCB detection in CSF). According to our results, no correlation was detected between the 6 studied cytokines and the VitD levels in the serum of MS patients, which could be in part attributed to the relatively limited size of the MS subgroup. As mentioned below, it is known that VitD plays multiple roles in the regulation of adaptive and innate immunity (expression of VDR and activating enzyme (CYP27B1) in different immune cells) [3]. Extensive murine studies and several ex vivo studies with primary human cells demonstrated that the active form of this vitamin may suppress pro-inflammatory cytokines (such as IL6, IL8, and TNFα) and enhance anti-inflammatory cytokines (such as IL10) [62]. It has been also demonstrated that there is a direct correlation between T regulatory cell percentages and 1, 25-(OH)2 vitD/25-OH ratios in MS patients [63]. The exact regulatory mechanism of VitD for innate cytokine production that leads to subsequent adaptive immune responses is still unknown.

Interestingly, we found that the level of IL6 was in positive correlation with IgG levels in serum. This cytokine was also positively correlated with the levels of the 5 other inflammatory cytokines (TNF, IL10, IL8, IL12p70, and IL-1β) in the serum of MS patients. Indeed, IL-6 is a pleiotropic cytokine produced by different types of cells: activated monocytes and T-cells, endothelial cells, and also residential cells in CNS (astrocytes and glial cells). Its wide range of effects (T-cell activation and differentiation, CTL differentiation to perforin production) includes an action on the immune humoral response: in fact, IL6 promotes B cell differentiation into Ig-secreting cells. This could be an explanation for our findings revealing a correlation of IL6 levels with IgG levels in serum during MS [64] [65]. The wide contribution of IL-6 in MS inflammatory processes was confirmed by its increase in serum and notably in CSF during MS. Its relation to disease severity has been also reported [64].

On the other hand, the mean value of TNFα was significantly higher in OCB positive (15.57 pg/mL) in comparison with OCB negative MS patients (9.2 pg/mL); Mean values of IL6, IL10 and IL1β were also higher when OCB are positive in CSF, but without a statistical significance. In fact, the contribution of TNF-α in MS pathogenesis may include an effect on the blood-brain barrier by increasing the endothelial permeability, in addition to the involvement of this cytokine in the demyelination process and oligodendrocyte damage [13]. Regarding the possible relationship between TNF-α and the intrathecal synthesis of Immunoglobulin (revealed as OCB) in our MS patients, it has been demonstrated that, during MS disease, B cells and/or plasma cells produce pro-inflammatory cytokines (IL-6, LTα, and GM-CSF), including TNF-α, with a deficient production of anti-inflammatory cytokines. Such B cell pro-inflammatory imbalance within the CNS may lead to CNS-compartmentalized inflammation, and subsequent intrathecal humoral immune response [66].

Regarding the clinical disability in MS, there was no correlation of any of the studied markers (cytokines, VitD) with EDSS score in our patients. These findings are consistent with the results of previous studies, where most measured cytokines (TNFα, IL10, IL6, IL2-R, and IL-1β) were reported to not correlate with this score during MS. It has been suggested that immunological activity (reflected by cytokine production) may be not interrupted in clinically stable patients [60].

## Conclusion

Our results support the association of VitD deficiency with MS, especially in female patients. These findings argue for the immunomodulatory effect of this sun-induced molecule and suggest its involvement, as a modifiable environmental factor, in disease development. However, the optimal range of vitD levels which should be targeted to have these regulatory roles on the immune system has not been clinically established. Despite the lack of correlation between vitD level and clinical disability in our study, it may be reasonable to study the potential effect of vitamin supplementation, as add-on therapy, on disease progression. Furthermore, in MS patients, the level of vitD in blood did not correlate with levels of circulating inflammatory cytokines. Interestingly, TNFα and IL6 levels were correlated with the intrathecal synthesis of IgG and the circulating IgG level, respectively, which could be explained by the involvement of these cytokines in B cell pro-inflammatory imbalance and the subsequent humoral response, especially in CNS. Larger studies analyzing comprehensive cytokine profiles in patients with MS are needed. The molecular and cellular basis of VitD effect on MS disease needs also to be clarified.

## Dodatak

### Compliance with ethical standards

#### Funding

This research did not receive any specific grant from funding agencies in the public, commercial, or not-for-profit sectors.

#### Ethical approval

All procedures performed in this study involving human participants were in accordance with the ethical standards of the institutional and national research committee.

### Conflict of interest statement

All the authors declare that they have no conflict of interest in this work.
